# Decreased miR-192 expression in peripheral blood of asthmatic individuals undergoing an allergen inhalation challenge

**DOI:** 10.1186/1471-2164-13-655

**Published:** 2012-11-21

**Authors:** Masatsugu Yamamoto, Amrit Singh, Jian Ruan, Gail M Gauvreau, Paul M O'Byrne, Christopher R Carlsten, J Mark FitzGerald, Louis-Philippe Boulet, Scott J Tebbutt

**Affiliations:** 1 UBC James Hogg Research Centre, St. Paul’s Hospital, University of British Columbia, Room 166, Burrard Building, 1081 Burrard Street, Vancouver, BC V6Z 1Y6, Canada; 2 Institute for HEART+LUNG Health, Vancouver, British Columbia, Canada; 3 Department of Medicine, Division of Respiratory Medicine, UBC, Vancouver, British Columbia, Canada; 4 Vancouver Coastal Health Research Institute, Vancouver General Hospital, Vancouver, British Columbia, Canada; 5 NCE CECR PROOF Centre of Excellence, Vancouver, British Columbia, Canada; 6 Department of Medicine, McMaster University, Hamilton, Ontario, Canada; 7 Centre de Pneumologie de L’Hopital, Université Laval, Sainte-Foy, Quebec, Canada

**Keywords:** Allergen inhalation challenge, Allergy, Asthma, Blood cells, Hsa-miR-192, MicroRNAs, NanoString nCounter assay

## Abstract

**Background:**

MicroRNAs are small non-coding RNAs that regulate gene expression at the post-transcriptional level. While they have been implicated in various diseases, the profile changes in allergen inhalation challenge are not clarified in human. We aimed to evaluate changes in the microRNA profiles in the peripheral blood of asthmatic subjects undergoing allergen inhalation challenge.

**Results:**

Seven mild asthmatic subjects participated in the allergen inhalation challenge. In addition, four healthy control subjects (HCs) were recruited. MicroRNA profiles in peripheral blood samples (pre-challenge and 2 hours post-challenge) were measured by the NanoString nCounter assay to determine changes in miRNA levels as these asthmatic subjects underwent an allergen inhalation challenge. One common miRNA, miR-192, was significantly expressed in both comparisons; HCs vs. pre-challenge and pre- vs. post-challenge, showing that miR-192 was significantly under-expressed in asthmatics compared to HCs and decreased in post-challenge at an FDR of 1%. Cell-specific statistical deconvolution attributed miR-192 expression in whole blood to PBMCs. MiR-192 was technically validated using real-time reverse transcription-quantitative polymerase chain reaction (RT-qPCR) showing that the level in asthmatics (pre-challenge) was significantly lower than HCs and that post-challenge was significantly lower than pre-challenge. The normalized relative miR-192 expression quantified using RT-qPCR specific to PBMCs was also validated. Ontology enrichment and canonical pathway analyses for target genes suggested several functions and pathways involved in immune response and cell cycle.

**Conclusions:**

The miRNA profile in peripheral blood was altered after allergen inhalation challenge. Change in miR-192 levels may be implicated in asthma mechanisms. These results suggest that allergen inhalation challenge is a suitable method to characterize peripheral miRNA profiles and potentially elucidate the mechanism of human asthma.

## Background

Exposure to allergen is one of the important factors to induce and enhance asthma pathogenesis, which is characterized by reversible bronchoconstriction, airway hyperresponsiveness and airway inflammation. Allergen inhalation challenge is a useful model to investigate the pathogenesis of allergic airway diseases [1]. The asthmatic response can be evaluated by airflow limitation which can be measured as forced expiratory volume in 1 second (FEV_1_) drop using a spirometer. Recently, we have reported differential changes in the gene expression of peripheral blood leukocytes in atopic asthmatic individuals 2 hours following allergen inhalation challenge [2], indicating the application of gene expression in blood for assessing the asthmatic response. As blood is the pipeline of the immune system, assessing changes in expression profiles gives a comprehensive view of the status of the immune system in health and disease [3]. Since allergen exposure triggers immune responses, including the production of various mediators, inflammatory cell proliferation and infiltration in the human body, the immune cells and their gene expression can be good exploratory targets to understand the mechanisms of the asthmatic response to allergen inhalation challenge.

MicroRNAs (miRNAs) are a class of small noncoding RNAs with a regulatory function on gene expression to control various biological processes such as cellular proliferation, apoptosis and differentiation. They have been implicated in the pathogenesis of malignancy, cardiovascular and other diseases [4]. Several studies have previously reported the role of miRNAs in asthma using the allergen inhalation challenge model. In the lung, miRNA profiles were significantly changed in the experimental mouse model mimicking acute and chronic human asthma [5,6]. In humans, while a study comparing the miRNA profile of airway biopsies revealed no significant difference in the expression of 227 miRNAs between mild asthmatics compared to healthy volunteers [7], a more recent study on cultured human bronchial epithelial cells revealed inherent differences in the expression of miRNAs isolated from healthy and asthmatic subjects [8]. Changes in miRNA profiles have not been clarified in the human asthmatic response during the allergen inhalation challenge. In this study, we focused on the miRNA profiles in human peripheral blood and hypothesized that miRNA profiles altered by allergen inhalation challenge can be detected in peripheral blood.

## Results

### Subjects characteristics

Seven subjects with stable, mild atopic asthma participated in the allergen inhalation challenge. Four healthy control subjects (HCs) were recruited to serve as controls. The demographics of seven subjects with mild atopic asthma and four HCs are presented in Table 1. All asthmatic subjects developed an immediate drop in FEV_1_ of greater than 20% (Additional file 1: Figure S1).

**Table 1:** Demographics of subjects

**Subject**	**Age (yr)**	**Sex (M:F)**	**Allergen**	**Pre PC**_**20**_**(mg/ml)**	**Post PC**_**20**_**(mg/ml)**	**Allergen induced shift (pre PC**_**20**_**/post PC**_**20**_**)**
**Asthmatics**						
**1**	28	F	Cat Pelt	12.8	ND	ND
**2**	34	F	Cat Pelt	2.7	6.1	0.44
**3**	27	M	Cat Pelt	4.5	1.8	2.5
**4**	42	F	Cat Hair	5.3	8.6	0.62
**5**	23	F	Cat Hair	0.3	0.2	1.5
**6**	26	F	Cat Hair	5.1	1.5	3.4
**7**	49	F	Cat Hair	3.6	1	3.6
**Mean ±SE**	32.7±3.3			3.3^†^	1.7^†^	2.0±0.5
**Healthy controls**						
**1**	33	F	ND			
**2**	43	F	ND			
**3**	21	M	ND			
**4**	43	M	ND			
**Mean ±SE**	35.0±4.5					

ND - Not determined, SE: standard error of the mean, †: geometric mean (PC_20_ values are measured on a log scale).

### Processing of peripheral blood for miRNA Panel Codeset

Peripheral blood was drawn immediately before (pre) and after allergen inhalation (post) from asthmatics. Peripheral blood from HCs was collected as controls. Extracted RNA was analysed using NanoString nCounter assay. One hundred and sixty-three of the 734 miRNAs profiled using the NanoString nCounter assay were above background across all samples. The data set was normalized to the sum of the 163 miRNAs, such that each sample had the same total miRNA code count. MiRNAs above at least 100 code counts were retained for downstream analysis. The dataset (72 miRNAs for 18 samples) underwent log_2_ transformation prior to statistical analysis.

### Differentially expressed miRNA in NanoString nCounter assay

**Figure 1:**
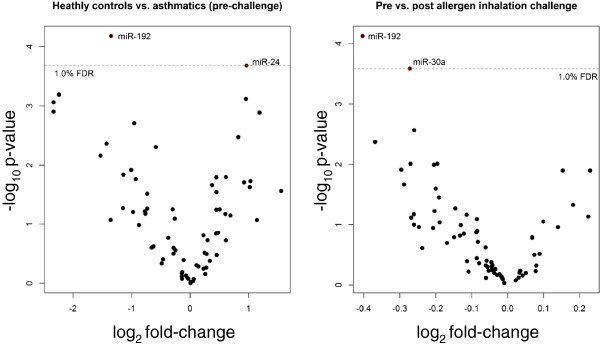
**Volcano plot of statistical significance against fold-change of differentially expressed miRNAs between healthy controls and asthmatics (pre-challenge) and between pre and post-challenge.** Dashed line represents a false discovery rate (FDR) of 1%.

**Figure 2:**
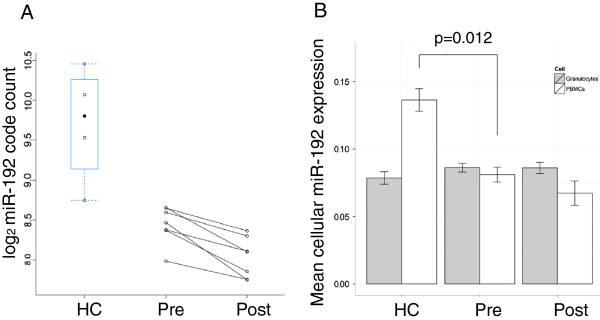
**MiR-192 expression in whole blood and in peripheral blood mononuclear cells (PBMCs). A**. MiR-192 expression in whole blood. **B**. Cell-specific miR-192 expression in granulocytes (gray bar) and PBMCs (white bar) comparing healthy control (HC) and asthmatics pre and post-challenge (Pre, Post).

Two independent linear models were used to determine significant miRNAs for the two comparisons; HCs versus pre-challenge, and pre versus post-challenge. MiR-192 was significant in both comparisons at a false discovery rate (FDR) of 1% (Figure 1). MiR-192 was down-regulated in both comparisons (Figure 2A), that is, miR-192 was significantly under-expressed in asthmatics (pre-challenge) compared to HCs and decreased following allergen inhalation challenge.

### MiR-192 expression in peripheral blood mononuclear cells (PBMCs)

Given the heterogeneous nature of whole blood, changes in miRNA expression may not be due to changes in miRNA expression in specific cells but due to changes in the relative cell-type frequencies. Leukocyte differentials were significantly different among the three groups, while complete blood counts such as erythrocytes, total leukocytes and platelets were not significantly different among groups (Table 2). In order to determine whether miR-192 expression was associated with certain cell-type frequencies, a multiple regression of miR-192 expression onto the cell-type frequencies was performed for each group (HC, pre and post) independently. Given the small sample size of HCs (n=4), the neutrophils, eosinophils and basophils were combined (added) to form a granulocyte group whereas the lymphocytes and monocytes were combined into a peripheral blood mononuclear cells (PBMCs) group. The regression coefficients representing the mean miR-192 expression for granulocytes and PBMCs were extracted for each group (Additional file 1: Figure S2). In order to determine whether the partials for granulocytes and PBMCs were significantly different between groups, a test-statistic for each cell-type between two independent groups was calculated (see Methods). This statistic compares whether the slopes for two independent groups are different for the same cell-type: that is, for the same increase in a particular cell frequency, is the increase in the mean miRNA expression greater in one group that the other. This increase is independent of changes in other cellular frequencies. MiR-192 expression at the same frequency of granulocytes is similar between HCs and asthmatics (pre- and post-challenge), however, the mean miR-192 expression was significantly (p=0.012) higher in HCs than in asthmatics (pre-challenge) for the same frequency of PBMCs independent of the frequency of granulocytes (Figure 2B). Additional file 1: Figure S3 shows the empirical distributions for these comparisons for 1000 permutations. This may suggest that the decrease in miR-192 seen in whole blood (Figure 2A) may be due to a decrease in miR-192 expression in PBMCs independent of changes in the frequency of granulocytes. Although Figure 2B shows that miR-192 expression in PBMCs decreases post-challenge which is also seen in whole blood (Figure 2A), this change was not significant.

**Table 2:** Complete blood count and leukocyte differentials for two comparisons

	**Healthy controls**	**Pre-challenge**	**Post-challenge**	**P-value HC vs. pre**	**P-value pre vs. post**
Complete blood count					
Erythrocytes (10^12^/L)	4.41 (0.17)	4.36 (0.08)	4.40 (0.08)	0.786	0.361
Platelets (10^9^/mL)	246 (18)	258 (37)	273 (36)	0.759	0.084
Leukocytes (10^9^/mL)	5.48 (0.84)	6.10 (0.35)	6.91 (0.37)	0.199	0.071
Leukocyte differentials					
Neutrophils (%)	56.4 (7.8)	57.9 (4.5)	65.0 (2.6)	0.782	0.012*
Lymphocytes (%)	29.6 (5.5)	29.5 (4.4)	25.6 (2.6)	0.989	0.120
Monocytes (%)	4.5 (0.7)	7.4 (0.7)	5.7 (0.8)	0.042*	0.013*
Eosinophils (%)	2.1 (0.6)	4.7 (0.8)	3.3 (0.7)	0.077	0.002*
Basophils (%)	5.6 (5.5)	0.5 (0.1)	0.4 (0.1)	0.002*	1.000

HC, healthy controls. pre, pre-challenge (asthmatics). post, post-challenge (asthmatics). Values reported as mean (standard error of the mean). *: p < 0.05.

### Technical validation using real-time reverse transcription-quantitative polymerase chain reaction (RT-qPCR)

**Figure 3:**
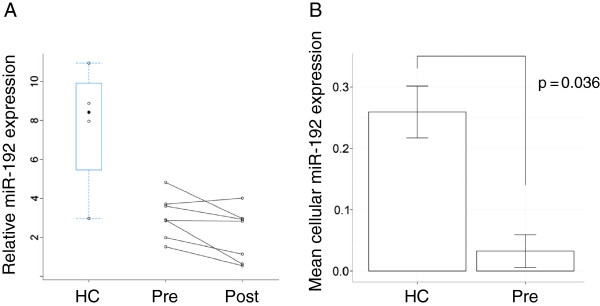
**Technical validation of miR-192 expression in whole blood and PBMCs. A**. MiR-192 expression quantified using RT-qPCR in whole blood. **B**. MiR-192 expression in PBMCs comparing healthy control (HC) and asthmatics (pre-challenge).

MiR-192 was technically validated (Figure 3A) using RT-qPCR showing that the level in asthmatics (pre-challenge) was significantly (p < 0.05) lower than HCs and that post-challenge was significantly lower than pre-challenge.

The normalized relative miR-192 expression quantified using RT-qPCR was also regressed onto the relative cell-type frequencies of granulocytes and PBMCs for HC and pre-challenge in separate linear models. The regression coefficient for PBMCs in healthy individuals (i.e. miR-192 expression in PBMCs in HC) was significantly (p=0.036) higher than the regression coefficient for PBMCs in asthmatics at pre-challenge (i.e. miR-192 expression in PBMCs in asthmatics at pre-challenge) (Figure 3B).

### Genes up-regulated in allergen inhalation challenge and targeted by miR-192

To clarify the suggestive mechanisms of miR-192 in allergen inhalation challenge, genes targeted by miR-192 were retrieved from the list of differentially expressed genes between pre- and 2 hour post-allergen challenge, which Kam *et al.* reported in their manuscript [2]. Among 80 genes which were significantly altered and predicted as target genes for miR-192 by databases miRanda and TargetScan, 56 genes were up-regulated while 24 genes were down-regulated post-challenge. To clarify the biological mechanisms, we performed network modeling using MetaCore from GeneGo (St Joseph, MI, USA), allowing us to build a candidate network indicating possible interactions between genes. The Functional Ontology Enrichment tool, which was used for analysing 56 up-regulated genes by considering their mappings onto terms of a given MetaCore ontology, showed a number of significant (p<0.01) inflammatory pathways such as ChREBP regulation pathway and IFNα/β signalling pathway (Additional file 1: Table S1). Similarly, the list of up-regulated target genes was used to generate biological networks using the Canonical Pathway Modeling algorithm. Additional file 1: Table S2 shows the list of networks that are highly enriched with canonical pathways. The results comprise network objects corresponding to genes and other interacting objects in canonical pathways found in databases. For the top two highly enriched pathways, one pathway was related to DNA damage and cell cycle regulation (Additional file 1: Figure S4A) and the other pathway was related to immunological response and stress response (Additional file 1: Figure S4B).

## Discussion

We analysed for the first time the change in miRNA profiles of human asthmatics undergoing allergen inhalation challenge as well as the difference in miRNA profiles between pre-level in asthmatics and healthy control subjects without asthma history. MiR-192 was significantly lower in post-allergen inhalation challenge compared to pre-challenge, as well as lower in asthmatics compared to healthy control subjects, suggesting that the change in miR-192 level may be involved in the atopic asthma and asthmatic reaction after allergen inhalation challenge. Cell specific expression of miR-192 was associated with peripheral blood mononuclear cells (PBMCs). Among the genes we have previously reported to be altered following allergen inhalation challenge [2], predicted target genes of miR-192 were largely up-regulated suggesting the inhibitory role of miR-192 on their expression. The result showing that these target genes were enriched for the functions on cell cycle and immune response supports the notion that miRNA can regulate such biological functions in the asthmatic response.

MiR-192 has been studied in various conditions including cancer and autoimmune diseases. Several reports showed that miR-192 affects cellular proliferation through the p53 pathway, which regulates cell cycle. The cell cycle checkpoint control genes, p53 and p21 were overexpressed in cells with overexpressed miR-192 *in vitro* using human cell lines [9]. In the canonical pathway modeling in our results for the up-regulated target genes for miR-192, cell cycle regulation and response to DNA damage was one of the top-listed pathways, suggesting that miR-192 mediates cell cycle regulation of blood cells in response to allergen challenge. In the other study investigating the response of miRNA to environment, exposure to cigarette smoke decreased miR-192 expression in the lung in animal experimental model [10]. Given that the cigarette smoke exposure induces airway inflammation and cellular stress such as oxidative stress, this report supports our findings that miR-192 is regulating the response to miRNAs to environmental exposure inducing airway inflammation. As shown in the other top-listed pathway in the canonical pathway modeling in our data, miR-192 was suggested to mediate the immune response following allergen inhalation. In addition, as a biomarker in peripheral blood, miR-192 has been reported to decrease in systemic lupus erythematosus, a systemic autoimmune disease inducing inflammatory responses [11]. Perturbation of miRNA profiles in response to inflammatory stimuli can occur in peripheral blood and the changes can be detected. Interestingly, miR-192 expression is also reportedly decreased in response to TGF-β and loss of miR-192 correlates with tubulointerstitial fibrosis and reduction in renal function in renal biopsies from patients with established diabetic nephropathy [12]. Allergen inhalation challenge induces up-regulation of TGF-β in the airway epithelium [13]. TGF-β has been implicated in airway remodeling and inflammation, which are features of chronic asthma. Although the origin of the miRNA needs to be clarified, our data showing down-regulated miR-192 in the blood after allergen inhalation challenge may indicate similar TGF-β derived mechanisms. Since the mechanism of miR-192 has not been elucidated in allergic airway diseases, further studies are needed to clarify these mechanisms.

Peripheral blood consists of various types of cells such that the expression of a given miRNA is the net expression from all the various cell-types. In this report, statistical methods for deconvolving cell-specific miRNA levels from whole blood experiments were used. This approach called cell-specific significance analysis of microarrays (csSAM) [14] can combine information from complete blood cell count, including leukocyte differentials, and whole blood gene expression data to deconvolve cell specific expression measurements that can then be compared across groups. We have previously shown that csSAM can uncover cell-specific gene expression signatures in whole blood in two independent studies [15,16]. We utilized this approach to analyse cell-specific analysis for miR-192 expression in granulocytes and PBMCs. The difference of miR-192 levels is suggested to be derived from PBMCs in our data, suggesting that further studies on the subtype of lymphocytes and monocytes will help reveal the mechanisms of miRNA in asthma and asthmatic responses as well. MiR-192 levels are reportedly different among subsets of lymphocytes [17]. A study comparing miRNA expression in a wide range of haematological cell lines showed that miR-192 was up-regulated in activated B cells [18]. Allergen challenge induces a dynamic shift of lymphocyte populations in blood. 24 hours after challenge there was a reduction of peripheral blood CD4^+^ T lymphocytes from a baseline whereas CD4^+^ T lymphocytes in bronchoalveolar fluid increased, suggesting lymphocyte recruitment into the respiratory system after allergen challenge [19,20]. In a baseline comparison between asthmatics and healthy controls, while there was no significant difference in number of T cells or B cells in peripheral blood [21,22], their subpopulations and activation state have been reported to be different. Several studies investigating peripheral blood cells in mild asthma also showed the increase in the number of activated population such as CD23-bearing B cells and CD25-bearing T cells in mild atopic asthmatics as well as low number of gamma delta T cells in the peripheral blood of asthmatics [22,23]. Given that cell populations and subpopulations can affect the miRNA levels, it is necessary to clarify the relationship between such cellular populations and differential miRNA profiles, for example through the use of statistical deconvolution methods such as csSAM.

The csSAM test statistic which compares the mean miRNA expression for each cell type between two independent groups does not take into account the paired structure of our longitudinal study design. Since it is possible to achieve statistical significance with smaller treatment effects in a paired study design, using an unpaired test statistic may explain why the reduction of miR-192 expression in PBMCs post-challenge compared to pre-challenge was not statistically significant (Figure 2B). Accounting for the within individual variation through the use of a mixed-effects model or multilevel data analysis with a modification to the csSAM test statistic may help improve the statistical significance of the csSAM test statistic in longitudinal studies. The small number of subjects limited the number of cell-types for cell-specific analyses in this study. Future studies with larger sample size will enable us to further study the role of additional cell types. In addition we have not compared the phenotypes induced by allergen inhalation challenge such as early responders and dual responders. The current analyses comparing pre and post combined these phenotypes, which may serve as confounding factors. Given the sample size a technical validation with RT-qPCR was deemed appropriate. Given the course of allergen inhalation challenge, a longer time course study of miRNA levels in blood in both asthmatics and healthy subjects also needs to be explored. Collectively, a further study using a different cohort consisting of a large sample size is needed to validate the decrease in miR-192 levels in asthmatics after allergen inhalation challenge. Thereafter, cell-specific approaches for differential miRNAs will also validate our results as well as shed light on further mechanisms of miRNAs in certain cell types in peripheral blood cells.

## Conclusion

MiRNA profile changes can be detected in peripheral blood of asthmatic subjects undergoing allergen inhalation challenge and between healthy control and asthmatics. Among them, changes in miR-192 level may be involved in asthma mechanisms. These results indicate that allergen inhalation challenge can be a suitable model to explore miRNA profiles and help elucidate the mechanisms of allergic asthma in humans.

## Methods

### Subjects and allergen inhalation challenge

This study was approved by University of British Columbia-Providence Health Care Research Ethics Board with informed consents obtained in compliance with the local Research Ethics Boards. Seven subjects with stable, mild atopic asthma participated in the allergen inhalation challenge. Four HCs were recruited to serve as controls. All subjects were non-smokers, free of other lung diseases, and not pregnant. Diagnosis of asthma was based on the Global Initiative for Asthma criteria. Asthmatic subjects were diagnosed as mild allergic asthma, and only used intermittent short-acting bronchodilators for treatment of their asthma. Asthmatic subjects had a baseline FEV1 ≥70% of predicted, and the provocative concentration of methacholine required to produce a 20% decrease in FEV_1_ (PC_20_) was ≤16 mg/mL [24]. The methacholine inhalation challenge was conducted as described by Cockcroft [25,26]. Skin prick tests were used to determine allergies to cat, and the dose of cat extract for inhalation. Allergen challenges were conducted as triad visits. On the first and third days, subjects underwent methacholine challenges for assessments of airway hyperresponsiveness, and on the second day subjects underwent allergen inhalation challenges as described by O’ Byrne *et al*. [27], using extracts of cat pelt or hair. Asthmatic subjects were challenged with cat allergen to reduce confounding effects of different types of allergens. Blood was drawn immediately before (pre) and approximately 2 hours after allergen inhalation (post). Peripheral blood from HCs was collected in the morning (9 a.m.) to accommodate the time point comparing to pre-level of asthmatic subjects.

### Blood collection and RNA extraction

Peripheral venous blood samples were collected into BD Vacutainer plastic EDTA tubes (Becton, Dickinson and Company, Franklin Lakes, NJ, USA). One aliquot was processed for total and differential cell counts, and the other aliquot was frozen and stored at −80°C until RNA extraction. From thawed samples, total RNA containing miRNA was purified from 400 μL of whole blood according to manufacturer’s protocols using the RNeasy Mini Kit (Qiagen, Chatsworth, CA, USA). The yield and quality of RNA were assessed by NanoDrop 8000 Spectrophotometer (Thermo Scientific, Wilmington, DE, USA) and Agilent 2100 Bioanalyzer (Agilent Technologies, Santa Clara, CA, USA).

### NanoString nCounter assay

Comprehensive assay for miRNA expression was performed using nCounter® miRNA Expression Assay Kits (NanoString Technologies, Seattle, WA, USA) at NanoString Technologies. In this method, the novel technology which enabled multiplexed direct digital counting of RNA molecules [28] were applied to miRNAs with some modification. Briefly, to prepare a miRNA molecule for hybridization in the nCounter assay, a proprietary DNA sequence called miRtag is ligated to the mature miRNA using a bridging oligonucleotide (bridge). The miRtags for the human miRNA are ligated and bridges are purified in one simple multiplexed reaction. After removal of the bridge, the tagged mature miRNA is then hybridized to a colour-coded reporter probe and a biotinylated capture probe. The capture probe allows the complex to be immobilized for data collection with measurement of its colour code. A total of 734 human and human-associated viral miRNAs were simultaneously assayed.

### NanoString nCounter miRNA assay protocol

MiRNA assays were performed using 100 ng of total RNA following the standard nCounter miRNA Assay Protocol. Hybridizations were carried out by mixing 5 μl of each miRNA assay with 20 μl NanoString nCounter reporter probe and 5 μl capture probe (30 μl total reaction volume) and incubating the hybridizations at 65°C for 18 hours.

### Preprocessing of miRNA Panel Codeset

The nCounter assay for each sample consisted of six positive controls (0.125-128 fM), eight negative controls, five control mRNAs (ACTB, B2M, GAPDH, RPL19 and RPLP0) and 734 miRNAs. Prior to normalization several probes in the codeset required background subtraction (Additional file 1: Table S3). The probes for which the background subtraction calculation produced a negative number were set to 1 for simplicity. To account for slight differences in assay efficiency (hybridization, purification, and binding) the data was normalized to the sum of 6 positive RNA spike-in controls. For each sample, the mean plus 2 times the standard deviation of the 8 negative controls was subtracted from each miRNA count in that sample. Only miRNAs with non-negative counts across all samples were retained for downstream analysis.

### Technical validation using RT-qPCR

RT-qPCR was carried out using the TaqMan MiRNA Assay (Applied Biosystems, Foster City, CA) according to the manufacturer's protocol. RNA samples were measured in duplicates. The TaqMan MicroRNA Reverse Transcription Kit (Applied Biosystems) was used for the preparation of cDNA. Reverse transcription reactions were performed in a volume of 15 μl, and each reaction contained 10 ng of total RNA including miRNA. The PCR reaction mix consisted of the RT product, TaqMan 2X Universal PCR Master Mix and the appropriate 5X MicroRNA Assay Mix containing primers and probe for the miRNA of interest. All TaqMan assays were run in duplicate on an ABI Prism 7900, applying 40 PCR cycles. Ct values were calculated with the SDS software using automatic baseline settings. Ct values >35 were considered to be below the detection level of the assay. RNU44 and RNU6B were used for normalizing the expression level of selected miRNAs. The mean of Ct values was subtracted from the corresponding Ct value for the selected miRNAs resulting in the ΔCt value which was used for relative quantification of miRNA expression.

### Statistical analysis

Complete blood cell count and leukocyte differentials were compared among groups using analysis of variance.Moderated robust regression in the Linear Model for Microarrays (limma) library from bioconductor was used to determine statistically significant miRNAs using a Benjamini Hochberg FDR of 1% [29]. A p-value of 0.05 was used to determine significant changes in cell counts. Significant cell-specific miRNA expression was determined using methods as previously described [14]. For each group, regression of miRNA expression onto the relative cell-type frequencies was used to determine the mean miRNA expression for each cell-type:

1. Multiple regression of miRNA expression onto the relative cell-type frequencies for each group g.

$$y_{g}=\beta_{0}+\beta_{1g}x_{1g}+\cdots +\beta_{\mathrm{kg}}x_{\mathrm{kg}}+\varepsilon_{g}=X\beta +\varepsilon \text{,}$$

*β*_0*g*_ = 0; implies that at zero cell-type frequency there is zero miRNA expression.

*β*_*kg*_; increase in miRNA expression for 1% increase in the k^th^ cell-type frequency in group g (mean miRNA expression in the k^th^ cell-type in group g)

*y*_g_; vector of miRNA expression values for group g

*x*_kg_: vector of relative cell-type frequencies for the k^th^ cell-type for group g

2. Test statistic to determine significant changes in cell-specific miRNA expression.

Similar to the Wald test to determine significant covariates, the following test statistic was used to determine significant miRNA expression changes in the k^th^ cell between two groups.

Test statistic comparing group 1 and group 2 for the k^th^ cell-type:

$$\begin{array}{c} t_{k21}=\frac{{\hat{\beta }}_{k2}-{\hat{\beta }}_{k1}}{se{\left({\hat{\beta }}_{k}\right)}_{21}},\;\\ se{\left({\hat{\beta }}_{k}\right)}_{21}=\sqrt{\frac{n_{1}{\left[se\left({\hat{\beta }}_{k1}\right)\right]}^{2}+n_{2}{\left[se\left({\hat{\beta }}_{k2}\right)\right]}^{2}}{n_{1}+n_{2}}} \end{array}$$

The p-value for each test-statistic was calculated by generating an empirical distribution by recalculating test-statistics after reshuffling of class labels 1000 times. A p-value of 0.05 was deemed significant.

### Target prediction, gene ontology analysis and canonical pathway analysis

Target genes for miR-192 were predicted using databases, miRanda and TargetScan to list the targets identified by both. Then the target genes for miR-192 were selected out of 1595 differentially expressed genes, which were identified post-allergen inhalation challenge at an FDR of 5% by Kam *et al*. [2].

Signalling pathways and cellular processes for target genes, which were reportedly up-regulated in post-allergen challenge and predicted to be targeted by miR-192, were defined through GeneGo MetaCore databases: Functional Enrichment by Ontology and Canonical Pathway Modeling.

## Competing interests

The authors declare that they have no competing interests.

## Authors' contributions

GMG, PMO, CC, JMF, LPB, SJT participated in research design and provision of samples. MY, JR, LPB, SJT participated in the sample processing and following experiments. MY, AS, SJT conducted data analyses. MY, AS, CC, SJT participated in the writing of the paper. All authors read and approved the final manuscript.

## Availability of supporting data

Supplementary Tables and Figures are shown in Additional_file_1.pdf.

## Supplementary Material

Additional file 1 Supplementary Tables and Figures. Click here for file
