# Supplementary material for: Decreased miR-192 expression in peripheral blood of asthmatic individuals undergoing an allergen inhalation challenge
Source: BMC Genomics. 2012 Nov 21;13:655. doi: 10.1186/1471-2164-13-655 (PMC3598672; doi:10.1186/1471-2164-13-655)
Supplement: Additional file 1 — Supplementary Tables and Figures. [file 1471-2164-13-655-S1.pdf]

(Supplementary Figures and Tables)

Decreased miR-192 expression in peripheral blood of asthmatic individuals undergoing an allergen inhalation challenge

Masatsugu Yamamoto[1,2,3,4], Amrit Singh[1,2], Jian Ruan[1,2], Gail M. Gauvreau[5], Paul M. O'Byrne[5], Christopher R.Carlsten[2,3,4], J.Mark FitzGerald[2,3,4], Louis-Philippe Boulet[6], Scott J. Tebbutt[1,2,3]

[1]James Hogg Research Centre, St. Paul's Hospital, University of British Columbia, Vancouver, British Columbia, Canada

[2]Institute for HEART+LUNG Health, Vancouver, British Columbia, Canada

[3]Department of Medicine, Division of Respiratory Medicine, UBC, Vancouver, British Columbia, Canada; Canada

[4]Vancouver Coastal Health Research Institute, Vancouver General Hospital, Vancouver, British Columbia, Canada

[5]Department of Medicine, McMaster University, Hamilton, Ontario, Canada

[6]Centre de Pneumologie de L'Hopital, Université Laval, Sainte-Foy, Quebec, Canada

Table of Contents

Supplementary Table 1. Functional Ontology Enrichment analysis for 56 upregulated target genes for miR-192.

Supplementary Table 2. Network enriched for 56 upregulated target genes for miR-192.

Supplementary Table 3. Probes in the miRNA Panel Codeset requiring background subtraction of the raw data.

Supplementary Figure 1. Percent drop in FEV_1 over time in 7 asthmatic subjects.

Supplementary Figure 2. Partial slopes for both granulocytes and PBMCs for HC, pre-challenge and post-challenge asthmatic individuals.

Supplementary Figure 3. Empirical distribution of test statistics comparing HC and asthmatics (pre-challenge) for 1000 permutations (re-shuffling of class labels) for granulocytes and PBMC.

Supplementary Figure 4. Top-listed enriched networks for 56 upregulated target genes for miR-192.

Supplementary Table 1. Functional Ontology Enrichment analysis for 56 upregulated target genes for miR-192. The Functional Ontology Enrichment tool provided by MetaCore from GeneGo was used for analysing target genes for miR-192 by considering their mappings onto terms of a given MetaCore ontology. The significantly enriched ontologies at p value less than 0.05 are shown. p-value was calculated in the algorithm as the probability of a random intersection of different gene sets.

MetaCore ontology	p value
Transcription_ChREBP regulation pathway	0.000029
Immune response_IFN alpha/beta signaling pathway	0.000044
Development_A2A receptor signaling	0.000261
Beta-2 adrenergic-dependent CFTR expression	0.000860
G-protein signaling_Rap2A regulation pathway	0.001110
Reproduction_GnRH signaling	0.001190
Development_Alpha-1 adrenergic receptors signaling via cAMP	0.001391
Transcription_CREM signaling in testis	0.001869
Apoptosis and survival_Beta-2 adrenergic receptor anti-apoptotic action	0.002043
G-protein signaling_Ras family GTPases in kinase cascades (scheme)	0.002611
Development_Signaling of Beta-adrenergic receptors via Beta-arrestins	0.002611
G-protein signaling_G-Protein alpha-i signaling cascades	0.002815
Development_Regulation of CDK5 in CNS	0.003026
Neurophysiological process_Mu-type opioid receptor-mediated analgesia	0.003469
Immune response_Signaling pathway mediated by IL-6 and IL-1	0.003469
Cell cycle_Role of Nek in cell cycle regulation	0.003941
LRRK2 in neurons in Parkinson's disease	0.004188
Cytoskeleton remodeling_Thyroliberin in cytoskeleton remodeling	0.004188
Nicotine signaling in glutamatergic neurons	0.004188
Regulation of CFTR gating (normal and CF)	0.004188
Signal transduction_Erk Interactions: Inhibition of Erk	0.004442
Development_NOTCH1-mediated pathway for NF-KB activity modulation	0.004442
wtCFTR and deltaF508 traffic / Membrane expression (norm and CF)	0.004442
G-protein signaling_G-Protein beta/gamma signaling cascades	0.004442
Nitrogen metabolism	0.004703
Inhibitory action of Lipoxin A4 on PDGF, EGF and LTD4 signaling	0.004703
Development_Hedgehog and PTH signaling pathways in bone and cartilage development	0.004970
Nitrogen metabolism/ Rodent version	0.004970
G-protein signaling_G-Protein alpha-s signaling cascades	0.004970
Development_Lipoxin inhibitory action on PDGF, EGF and LTD4 signaling	0.004970
G-protein signaling_G-Protein alpha-12 signaling pathway	0.005245
Cytoskeleton remodeling_ACM3 and ACM4 in keratinocyte migration	0.005526
Signal transduction_cAMP signaling	0.005526
Development_PACAP signaling in neural cells	0.005814
Transcription_NF-kB signaling pathway	0.005814
Development_Role of Activin A in cell differentiation and proliferation	0.006109

Cytoskeleton remodeling_Role of PKA in cytoskeleton reorganisation	0.006109
Reproduction_Progesterone-mediated oocyte maturation	0.006109
Development_GH-RH signaling	0.006411
Neurophysiological process_Netrin-1 in regulation of axon guidance	0.006411
Transport_ACM3 in salivary glands	0.006719
Apoptosis and survival_BAD phosphorylation	0.006719
Neurophysiological process_HTR1A receptor signaling in neuronal cells	0.006719
Nicotine signaling in dopaminergic neurons, Pt. 2 - axon terminal	0.007034
Neurophysiological process_Melatonin signaling	0.007034
Neurophysiological process_PGE2-induced pain processing	0.007034
cAMP/ Ca(2+)-dependent Insulin secretion	0.007034
Immune response_IL-1 signaling pathway	0.007356
Regulation of lipid metabolism_Regulation of lipid metabolism by niacin and isoprenaline	0.007684
Neurophysiological process_Glutamate regulation of Dopamine D1A receptor signaling	0.007684
Development_Ligand-independent activation of ESR1 and ESR2	0.007684
Immune response_PGE2 signaling in immune response	0.007684
Neurophysiological process_ACM regulation of nerve impulse	0.008019
Development_Hedgehog signaling	0.008019
Development_G-Proteins mediated regulation MAPK-ERK signaling	0.008019
Nicotine signaling in chromaffin cells	0.008019
Immune response_MIF - the neuroendocrine-macrophage connector	0.008019
Neurophysiological process_Dopamine D2 receptor signaling in CNS	0.008360
Regulation of lipid metabolism_Insulin signaling:generic cascades	0.008360
Transcription_CREB pathway	0.008360
Development_Beta-adrenergic receptors regulation of ERK	0.008360
Development_Leptin signaling via PI3K-dependent pathway	0.008360
Development_PDGF signaling via MAPK cascades	0.008360
Transport_Alpha-2 adrenergic receptor regulation of ion channels	0.008360
Muscle contraction_Relaxin signaling pathway	0.008708
Development_Melanocyte development and pigmentation	0.009062
Development_A3 receptor signaling	0.009062
Apoptosis and survival_HTR1A signaling	0.009423
Neurophysiological process_GABAergic neurotransmission	0.009423
Neurophysiological process_Corticoliberin signaling via CRHR1	0.009423
Immune response_Histamine signaling in dendritic cells	0.009423
Development_A2B receptor: action via G-protein alpha s	0.009423
wtCFTR and delta508-CFTR traffic / Generic schema (norm and CF)	0.009423
Signal transduction_PKA signaling	0.009790
Development_Beta-adrenergic receptors signaling via cAMP	0.010160
ENaC regulation in airways (normal and CF)	0.010160
Immune response_PGE2 common pathways	0.010160
Development_A1 receptor signaling	0.010540
Immune response_HMGB1/RAGE signaling pathway	0.010540
Immune response_CD28 signaling	0.010930
Membrane-bound ESR1: interaction with G-proteins signaling	0.010930
PGE2 pathways in cancer	0.011320
Airway smooth muscle contraction in asthma	0.011720
Muscle contraction_Regulation of eNOS activity in cardiomyocytes	0.011720
Regulation of lipid metabolism_Insulin regulation of glycogen metabolism	0.011720
Cardiac Hypertrophy_Ca(2+)-dependent NF-AT signaling in Cardiac Hypertrophy	0.012120
Regulation of CFTR activity (norm and CF)	0.012530

Transcription_PPAR Pathway	0.013800
Development_Thyroliberin signaling	0.013800
Blood coagulation_GPCRs in platelet aggregation	0.018410
Regulation of lipid metabolism_Stimulation of Arachidonic acid production by ACM receptors	0.018900
Neurophysiological process_NMDA-dependent postsynaptic long-term potentiation in CA1 hippocampal neurons	0.023030
Muscle contraction_GPCRs in the regulation of smooth muscle tone	0.024670
Development_VEGF signaling via VEGFR2 - generic cascades	0.025220
Regulation of lipid metabolism_Insulin regulation of fatty acid methabolism	0.028080
Histidine-glutamate-glutamine metabolism	0.031680
G-protein signaling_Rap1B regulation pathway	0.032060
Cytoskeleton remodeling_RalB regulation pathway	0.037780
IL-1 beta-dependent CFTR expression	0.046300
Histidine-glutamate-glutamine and proline metabolism/ Rodent version	0.048440

Supplementary Table 2. Network enriched for 56 upregulated target genes for miR-192. Canonical pathway networks were enriched and listed by the Canonical Pathway Modeling algorithm. p-value was calculated in the algorithm as the probability of a random intersection of different gene sets. Each network is associated with a Z-score which ranks the networks according to saturation with the objects from the gene list of seed nodes. Network 1 and 2 are depicted in Supplementary Figure 4.

	Network	Gene ontology processes	p value	z Score
1	TOPBP1, PMS2, Jagged2, APC/CDC20 complex, BRIP1	cell cycle process (42.7%; 3.862×10^{-26}), DNA repair (31.5%; 8.225×10^{-26}), cell cycle (46.1%; 8.365×10^{-26}), M phase (33.7%; 1.388×10^{-25}), response to DNA damage stimulus (34.8%; 1.385×10^{-24})	1.840×10^{-128}	158.92
2	CD48, STAT1, NKp46, NKp30, KLRC4 (NKG2F)	regulation of immune system process (62.8%; 3.967×10^{-24}), immune system process (72.1%; 2.619×10^{-23}), regulation of immune response (51.2%; 2.505×10^{-21}), regulation of response to stimulus (74.4%; 4.572×10^{-20}), regulation of response to stress (51.2%; 3.620×10^{-19})	5.690×10^{-30}	63.48
3	Endoplasmin, eIF2S1, GRP78, JIK, S1P	activation of signaling protein activity involved in unfolded protein response (50.0%; 1.067×10^{-21}), positive regulation of nuclease activity (50.0%; 1.598×10^{-21}), regulation of nuclease activity (50.0%; 4.947×10^{-21}), cellular response to unfolded protein (50.0%; 1.707×10^{-20}), endoplasmic reticulum unfolded protein response (50.0%; 1.707×10^{-20})	1.730×10^{-6}	19.52
4	PRKAR1A, VEGF-D, L-Noradrenaline extracellular region, PLGF, HGF	enzyme linked receptor protein signaling pathway (71.4%; 3.580×10^{-17}), transmembrane receptor protein tyrosine kinase signaling pathway (66.7%; 4.948×10^{-17}), activation of protein kinase activity (47.6%; 4.106×10^{-13}), positive regulation of protein kinase activity (52.4%; 9.760×10^{-13}), regulation of body fluid levels (57.1%; 1.170×10^{-12})	1.520×10^{-6}	19.94
5	GABA-B receptor, GIRK, GABA-A receptor, SLC7A6, G-protein beta/gamma	synaptic transmission (88.9%; 2.398×10^{-11}), transmission of nerve impulse (88.9%; 6.014×10^{-11}), multicellular organismal signaling (88.9%; 6.235×10^{-11}), cell-cell signaling (88.9%; 5.913×10^{-10}), regulation of ion transmembrane transport (55.6%; 6.892×10^{-8})	1.640×10^{-2}	7.66

6	REPS2, GGTase-I, Ca('2+) cytosol, FPR, RIT	small GTPase mediated signal transduction (63.6%; 8.079×10^{-10}), enzyme linked receptor protein signaling pathway (72.7%; 8.713×10^{-10}), nerve growth factor receptor signaling pathway (54.5%; 1.792×10^{-9}), transmembrane receptor protein tyrosine kinase signaling pathway (63.6%; 7.000×10^{-9}), signal transduction (100.0%; 1.481×10^{-7})	1.160×10^{-2}	9.16

Supplementary Table 3. Probes in the miRNA Panel Codeset requiring background subtraction of the raw data. The number of counts to be subtracted for a given probe is determined by multiplying a correction factor by the number of counts observed for the 128 fM positive control in the same lane.

Example: Lane 1: 128fM positive control = 20,000 counts, hsa-miR-16 = 500 counts.

Corrected hsa-miR-16 = 500 - (20,000*0.01) = 300 corrected counts

Probe	Correction Factor
hsa-miR-27b	0.08
hsa-miR-133a	0.05
hsa-miR-544	0.03
hsa-miR-320c	0.03
hsa-miR-590-5p	0.01
hsa-miR-16	0.01
hsa-miR-494	0.01
hsa-miR-579	0.01
hsa-miR-193a-3p	0.005
hsa-miR-196a	0.005
hsa-miR-485-3p	0.005
hsa-miR-1283	0.005
hsa-miR-192	0.005

Supplementary Figure 1. Percent drop in FEV_1 over time in 7 asthmatic subjects. X axis represents the time point after allergen inhalation challenge (hour). Y axis represents percent drop in FEV_1 from baseline (%).

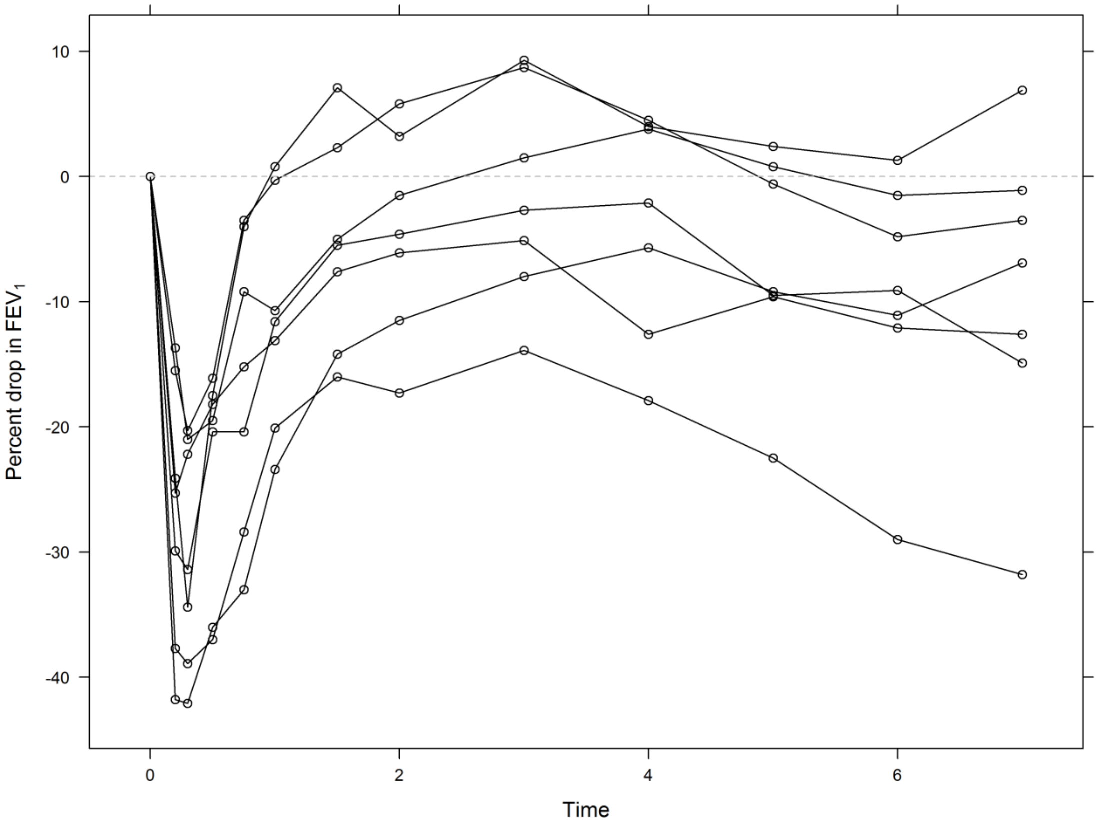

Supplementary Figure 2. Partial slopes for both granulocytes and PBMCs for HC, pre-challenge and post-challenge asthmatic individuals.

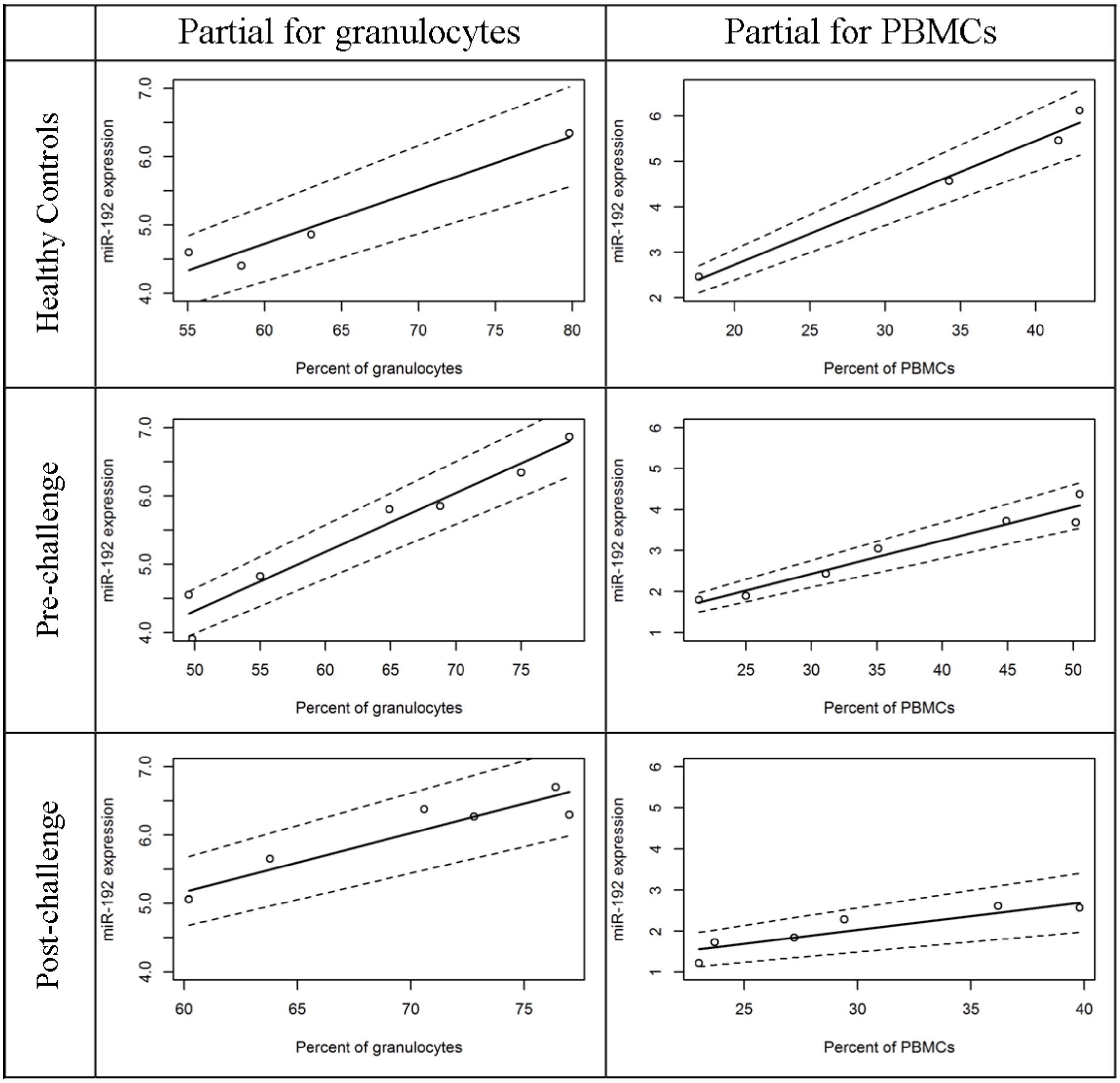

Supplementary Figure 3: Empirical distribution of test statistics comparing HC and asthmatics (pre-challenge) for 1000 permutations (re-shuffling of class labels) for granulocytes and PBMC. Red line indicates the test-statistic for the original comparison, and the dashed line indicates the significance level of 0.05.

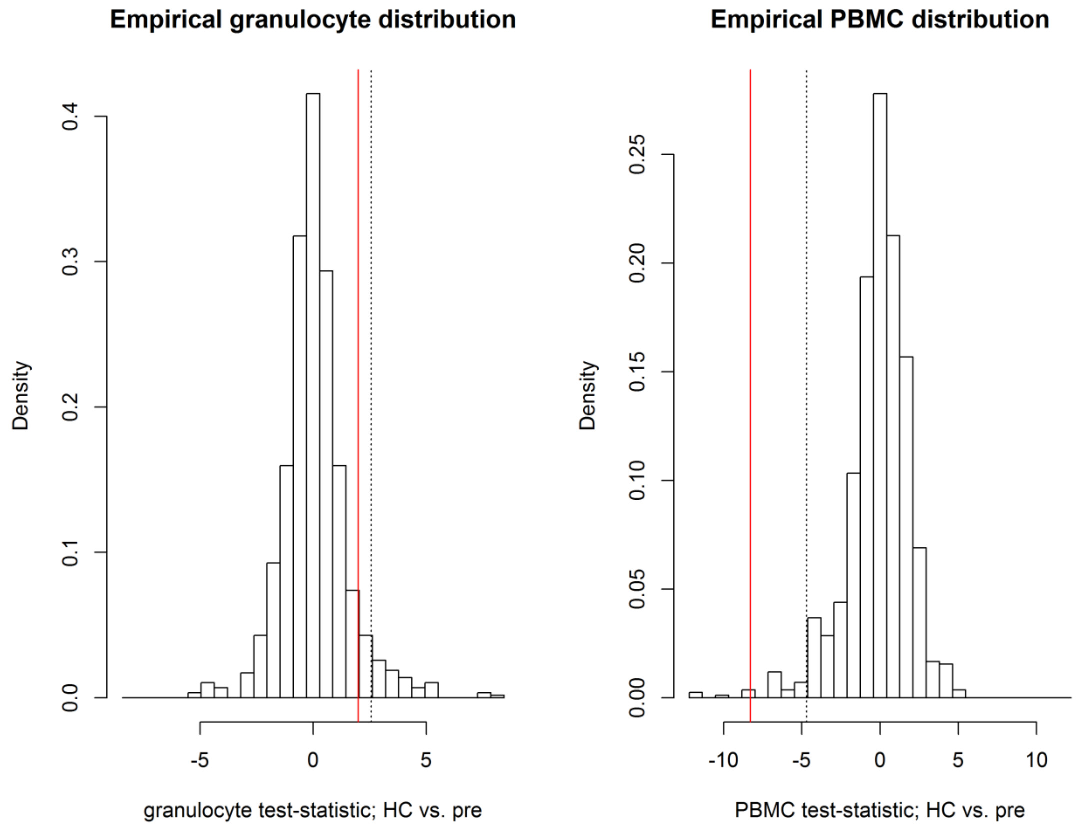

Supplementary Figure 4. Top-listed enriched networks for 56 upregulated target genes for miR-192. Most significant two canonical pathway networks are shown. Upregulated target genes are labeled with red circle. A: TOPBP1, PMS2, Jagged2, APC/CDC20 complex, BRIP1 pathway (Network 1 in Supplementary Table 2). B: CD48, STAT1, NKp46, NKp30, KLRC4 (NKG2F) pathway (Network 2 in Supplementary Table 2).

A

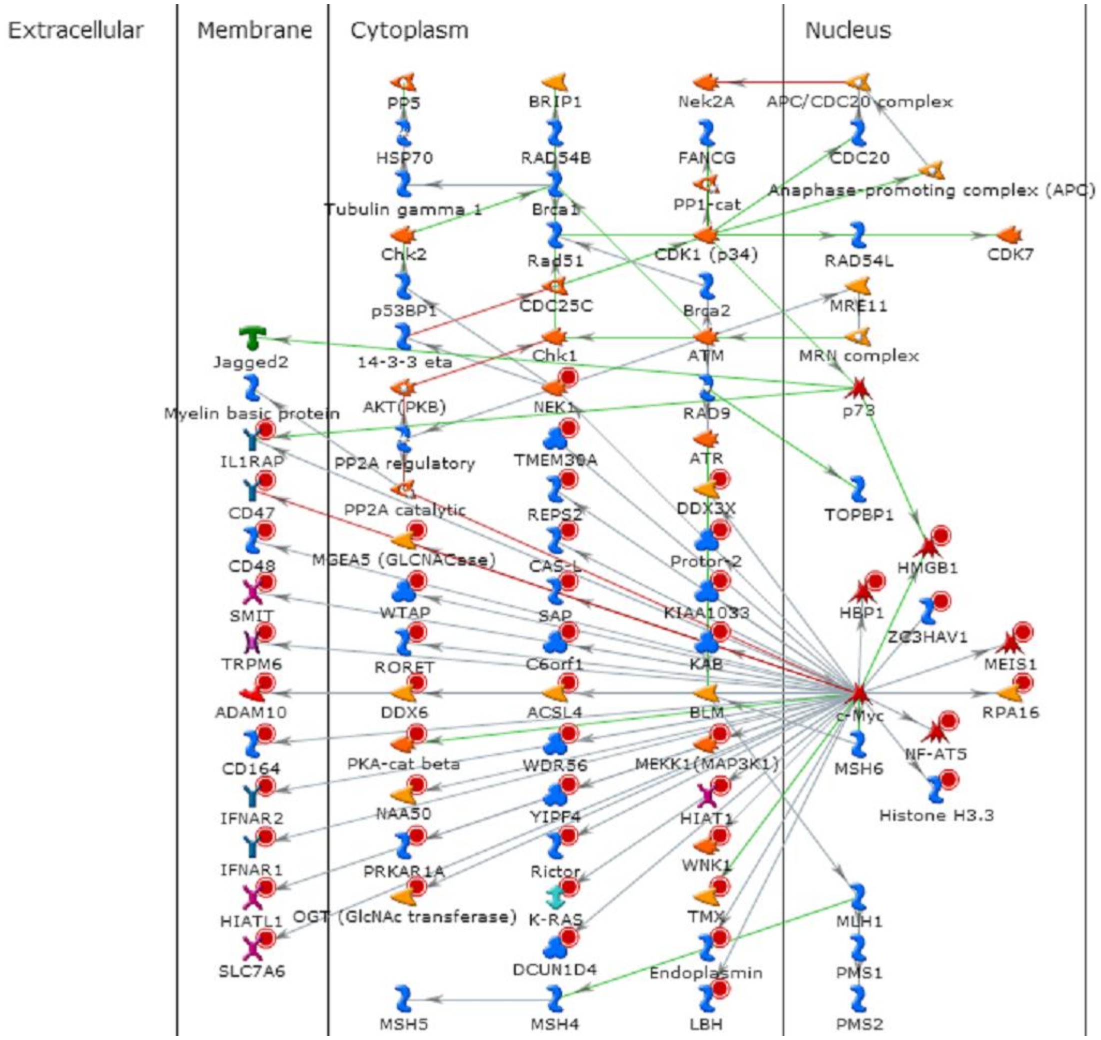

B

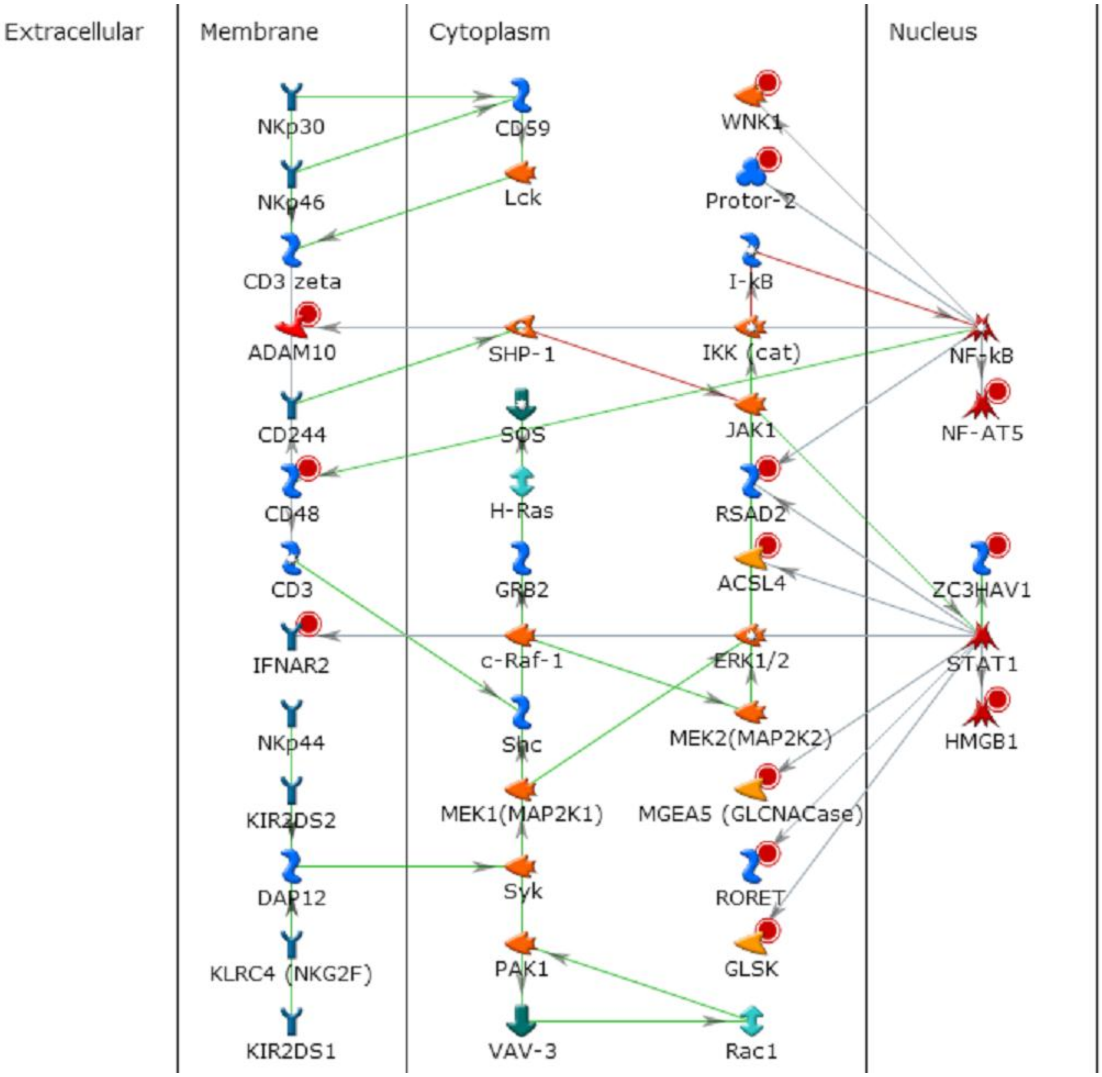

Extracellular
Membrane
Cytoplasm
Nucleus
NKp30
CD59
WNK1
NKp46
Lck
Protor-2
CD3 zeta
I-kB
ADAM10
SHP-1
IKK (cat)
NF-kB
CD244
SOS
JAK1
NF-AT5
CD48
H-Ras
RSAD2
CD3
GRB2
ACSL4
ZC3HAV1
IFNAR2
c-Raf-1
ERK1/2
STAT1
NKp44
Shc
MEK2(MAP2K2)
HMGB1
KIR2DS2
MEK1(MAP2K1)
MGEA5 (GLCNACase)
DAP12
Syk
RORET
KLRC4 (NKG2F)
PAK1
GLSK
KIR2DS1
VAV-3
Rac1
